# Distinct Requirements for CD4^+^ T Cell Help for Immune Responses Induced by mRNA and Adenovirus‐Vector SARS‐CoV‐2 Vaccines

**DOI:** 10.1002/eji.202451142

**Published:** 2024-11-27

**Authors:** Lyn Yong, Claire Hutchings, Eleanor Barnes, Paul Klenerman, Nicholas M. Provine

**Affiliations:** ^1^ Pandemic Sciences Institute Nuffield Department of Medicine University of Oxford Oxford UK; ^2^ Translational Gastroenterology and Liver Unit, Nuffield Department of Medicine–Experimental Medicine University of Oxford Oxford UK; ^3^ Peter Medawar Building for Pathogen Research, Nuffield Department of Medicine University of Oxford Oxford UK; ^4^ Centre for Human Genetics Nuffield Department of Medicine University of Oxford Oxford UK

**Keywords:** adenovirus vaccines, antibodies, CD4 T cell help, CD8 T cells, mRNA vaccines, T cell memory, T cell priming, vaccination

## Abstract

CD4^+^ T cells have been established as central orchestrators of cellular and humoral immune responses to infection or vaccination. However, the need for CD4^+^ T cell help to generate primary CD8^+^ T cell responses is variable depending on the infectious agent or vaccine and yet consistently required for the recall of CD8^+^ T cell memory responses or antibody responses. Given the deployment of new vaccine platforms such as nucleoside‐modified mRNA vaccines, we sought to elucidate the requirement for CD4^+^ T cell help in the induction of cellular and antibody responses to mRNA and adenovirus (Ad)‐vectored vaccines against SARS‐CoV‐2. Using antibody‐mediated depletion of CD4^+^ T cells in a mouse immunization model, we observed that CD4^+^ T cell help was dispensable for both primary and secondary CD8^+^ T cell responses to the BNT162b2 and mRNA‐1273 mRNA vaccines but required for the AZD1222 Ad‐vectored vaccine. Nonetheless, CD4^+^ T cell help was needed by both mRNA and Ad‐vectored vaccine platforms for the generation of antibodies, demonstrating the centrality of CD4^+^ T cells in vaccine‐induced protective immunity against SARS‐CoV‐2. Ultimately, this highlights the shared and distinct regulation of humoral and cellular responses induced by these vaccine platforms.

## Graphical abstract

The role of CD4^+^ T cells in promoting cellular and humoral immunity induced by novel mRNA–LNP and adenovirus vector vaccine technologies is incompletely understood. Surprisingly, we identified a discordant need for CD4^+^ T cell help based on both vaccine platform and arm of adaptive immunity examined. These data provide further evidence of the unique biology underpinning these new technologies.

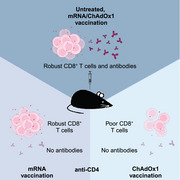

## 1 Introduction

CD4^+^ T cells are essential for the effective induction of both humoral and cellular immunity to many vaccines and infectious agents [1]. CD4^+^ T cells crosstalk with innate immune cells, providing the necessary signals—termed “help”—for further activation of downstream adaptive immune cells [1]. This can be through inducing expression of costimulatory molecules or the secretion of cytokines, providing the optimal environment for activation of other immune cells, including CD8^+^ T and B cells [1, 2]. Hence, CD4^+^ T cells are routinely viewed as central orchestrators of adaptive immunity. However, the role of CD4^+^ T cell help in promoting the cellular and humoral responses to the novel nucleoside‐modified mRNA–LNP (lipid nanoparticle) and adenovirus (Ad)‐vectored SARS‐CoV‐2 vaccines has not been thoroughly explored.

In many settings, CD4^+^ T cells are needed for generation of primary CD8^+^ T effector cell responses. CD4^+^ T cell “licensing” of antigen‐presenting cells (APCs) via CD40L and CD40 interactions increases antigen presentation, costimulatory molecule expression [3, 4] and production of chemokines [5] by APCs to promote CD8^+^ T cell effector responses. CD4^+^ T cell‐derived IL‐2 also aids the survival and proliferation of CD8^+^ T cells [6]. The necessity of such CD4^+^ T cell help is demonstrated in attenuated primary CD8^+^ T cell responses against vaccinia virus [7], influenza [8], herpes simplex virus [9], and in vaccination with Ad‐vectored vaccines [10–12] in the absence of CD4^+^ T cells. In contrast, the generation of primary CD8^+^ T cell responses to lymphocytic choriomeningitis virus (LCMV) [13] and *Listeria monocytogenes* [14] occurs independently of CD4^+^ T cell help. This discordance was partially resolved by a model in which certain viruses and bacteria were able to trigger strong Type I IFN responses from APCs, bypassing the need for CD4^+^ T cell help [15]. Given the specific engineering of the nucleoside‐modified mRNA–LNP vaccines to minimize induction of Type I IFN [16–18], this model would suggest a critical dependency on CD4^+^ T cell help.

CD4^+^ T cells were also demonstrated to be critical for memory CD8^+^ T cell responses, especially in their maintenance, and anamnestic potential to respond to secondary antigen exposure. Mechanisms include the downregulation of TRAIL‐mediated apoptosis of CD8^+^ T cells [19] and direct CD40 and CD40L interactions with CD4^+^ T cells [20]. Another study elucidated that IL‐2 during the primary response helps imprint the responsiveness of CD8^+^ T cells to secondary exposure [21]. The crucial role of CD4^+^ T cells in the expansion of memory CD8^+^ T cells and effective secondary responses is evident in models of influenza [22], Ad‐vectored vaccines [23], and critically even when such help was dispensable for the primary response, such as LCMV [13] and *L. monocytogenes* [24] infection.

Similarly, the requirement for CD4^+^ T cell help in the induction of effective antibody responses is well established, which is facilitated by the CD4^+^ T follicular helper (T_FH_) cell subset [1]. T_FH_ cells, through multiple mechanisms, including CD40 and CD40L interactions [25] and expression of ICOS [26], support germinal center (GC) formation to produce somatic‐hypermutated B cells, which produce neutralizing antibodies. This is evident in the need for T_FH_ cells in the induction of humoral responses to viral infections [27, 28] and Ad‐vectored vaccination [29]. However, the requirement of T_FH_ cells for antibody production to mRNA vaccines is unclear. One study has shown T_FH_ cells accumulate in lymph nodes following mRNA vaccination [30], but another suggested that neutralizing antibodies can be produced in their absence [31].

Given that the formal requirement for CD4^+^ T cell help to generate immune responses varies across viral infections and vaccines, we sought to determine the role of CD4^+^ T cells in the context of cellular and humoral responses to nucleoside‐modified mRNA and Ad‐vectored vaccines against SARS‐CoV‐2. This includes Pfizer‐BioNTech's BNT162b2 and Moderna's mRNA‐1273 mRNA vaccines, and Oxford‐AstraZeneca's AZD1222 (ChAdOx1 nCoV‐19) Ad‐vectored vaccine. CD4^+^ T cells were depleted a day prior to immunization in mice, and responses were quantified. We observed that CD4^+^ T cell help was dispensable for primary and secondary CD8^+^ T cell responses to mRNA vaccines. However, this was not a characteristic of the antigen as CD8^+^ T cell responses to AZD1222 were attenuated with CD4^+^ T cell depletion. On the other hand, CD4^+^ T cell help was indispensable for the induction of humoral responses to vaccination, irrespective of the vaccine platform. This demonstrates shared and distinct requirements for CD4^+^ T cell help in the context of humoral and cellular responses induced by the two vaccine technologies.

## 2 Materials and Methods

### 2.1 Mice and Tissue Processing

6‐week‐old female C57BL/6J mice from Charles River were housed in individually ventilated cages in specific‐pathogen‐free conditions at the Biomedical Sciences Building (University of Oxford). All procedures were conducted by licensed and trained individuals under UK Home Office project license PP3430109 in accordance with the UK Animals (Scientific Procedures) Act (1986).

Mice were immunized intramuscularly in the right hind leg with 5 × 10^9^ viral particles (vp) of AZD1222 or 1 µg of the mRNA vaccines mRNA‐1273 or BNT162b2. AZD1222 was produced by the Viral Vector Core Facility (University of Oxford), whereas the mRNA vaccines were kindly provided by the OUH Pharmacy as clinical waste material. Anti‐CD4 (clone GK1.5; BioXcell) was administered intraperitoneally 1 day prior to immunization at 500 µg/mouse. PBMCs were isolated by Lymphoprep density centrifugation at 1900 rpm for 20 min. Serum was isolated from the remaining blood by two sequential rounds of centrifugation at 5000 *g* for 5 min. Spleens and lymph nodes were harvested in 5% FBS‐RPMI and mechanically dissociated through 70 µm filters to obtain single‐cell suspensions. Splenic RBCs were lysed by 1 × RBC lysis buffer (BioLegend) solution for 3 min.

### 2.2 Flow Cytometry

Staining antibodies, clones, and concentrations are listed in Table S1. MHC Class I tetramer staining was carried out with H‐2K^b^ tetramers loaded with the SARS‐CoV‐2 S_539–546_ peptide (VNFNFNGL) [32]. Biotinylated monomers were provided by the NIH Tetramer Core Facility, and tetramers were prepared with phycoerythrin (PE)–streptavidin according to their instructions. The SARS‐CoV‐2 S1 recombinant protein tetramers were conjugated to PE or Brilliant Violet 421 and prepared as described in [33].

Surface staining was performed for 30 min at 4°C, and cells were washed, followed by fixation and permeabilization for 20 min at 4°C with Cytofix/Cytoperm (BD Biosciences). For intracellular staining, cells were stimulated with the S_538–546_ peptide [32] for 5 h at 37°C. Brefeldin A and anti‐CD107a (clone 1D4B) were added during peptide stimulation. Cells were then washed, surface stained, and permeabilized. Intracellular staining was performed for 30 min at 4°C, washed twice with 1 × Perm/Wash buffer (BD Biosciences) and stored in FACS (1 mM EDTA, 0.05% BSA in PBS) buffer until analysis on an LSRFortessa.

### 2.3 Quantification of Antigen‐Specific CD8^+^ T Cells

After spleens were processed, total cell numbers were counted on the LUNA‐FX7 automated cell counter (Logos Biosystems). The number of K^b^/S_539–546_^+^ CD8^+^ T cells in the spleen was then calculated by taking the frequency of K^b^/S_539–546_^+^ CD8^+^ T cells of total live lymphocytes and multiplying by the total number of splenocytes counted.

### 2.4 Quantification of Anti‐Spike IgG

Anti‐spike IgG from mouse sera was measured by enzyme‐link immunosorbent assay (ELISA) adapted from [34], where titers are a function of the number of dilutions to reach below twice the absorbance value of blank wells. Briefly, wells were coated with 1 µg/mL of S1 + S2 (BioLegend) proteins overnight at 4°C and blocked with 2% BSA 0.05% Tween 20‐PBS for 4 h at room temperature. Sera were added and incubated for 1 h. Washing with 0.05% Tween 20‐PBS was carried out thrice before adding HRP‐labeled anti‐mouse IgG antibody (Southern Biotech) at an 1:1000 dilution. After washing thrice with 0.05% Tween 20‐PBS, TMB was added and developed for 30 s and immediately read at 450 nm on a Biochrom EZ Read 400.

### 2.5 Data Analysis and Statistics

All flow cytometry data were analyzed on FlowJo v.10. All data were analyzed in GraphPad Prism v10.3.1. All statistical analyses displayed on the graphs are unpaired Student's *t*‐tests with a value of *p* < 0.05 considered statistically significant. Only statistically significant differences are shown. All gating strategies are shown in Figures S1–S3. For expression of cytokines, values from unstimulated samples were used as background that was subtracted from the values presented in the bar graphs. Polyfunctionality of CD8^+^ T cells was analyzed using Boolean combination gates in FlowJo software to combine any cell expressing CD107a, IFNγ, TNF, or IL‐2 into a population (cytokine^+^ CD8^+^ T cell population). The contribution of each marker of the cytokine^+^ CD8^+^ T cell population was computed with the SPICE program v6.1 to generate pie charts.

## 3 Results

### 3.1 CD4^+^ T Cell Help Is Dispensable for Generation of Antigen‐Specific CD8^+^ T Cell Responses to mRNA‐1273

**FIGURE 1:**
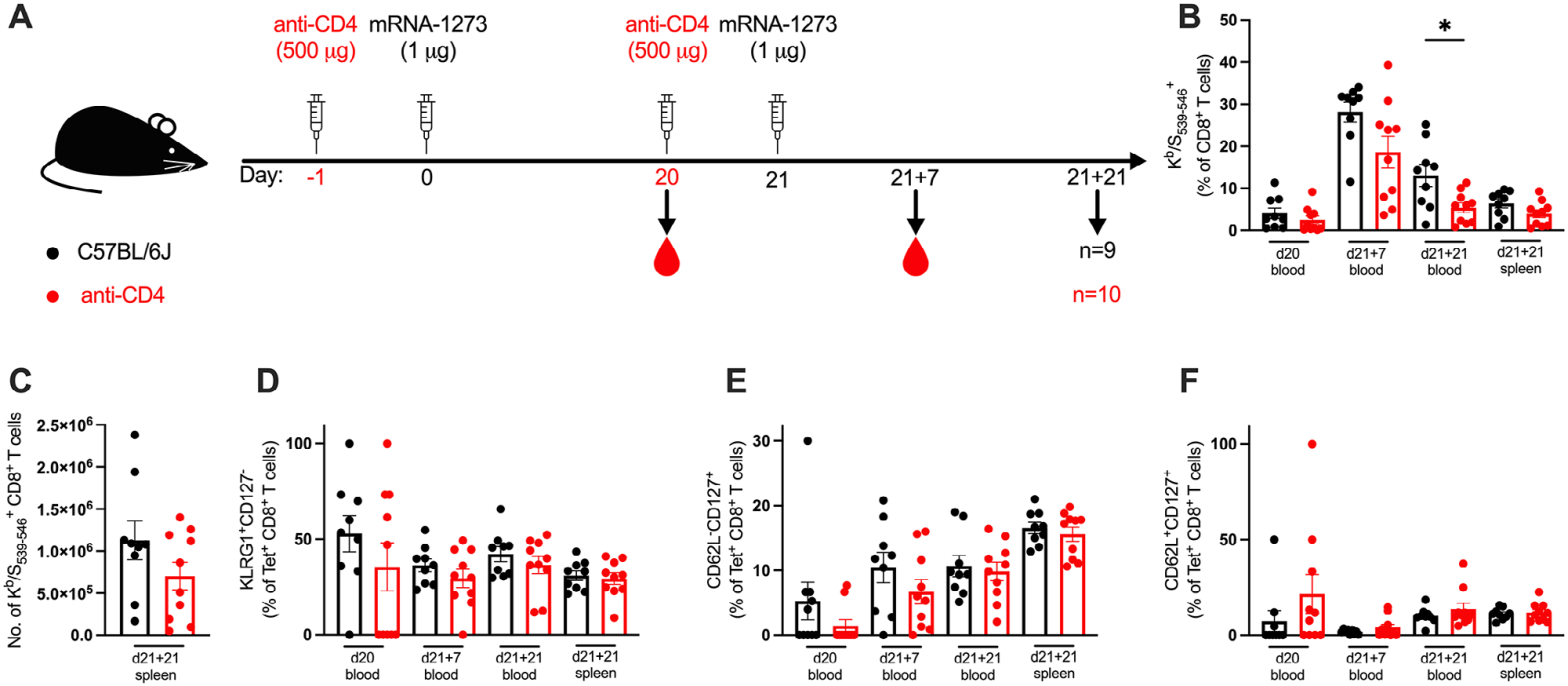
CD4^+^ T cell help is dispensable for generation of antigen‐specific CD8^+^ T cell responses to mRNA‐1273. (A) A schematic diagram illustrating the experimental setup: Anti‐CD4 antibody was administered intraperitoneally 1 day prior to each dose of mRNA‐1273. Cells from blood and spleen were isolated for analysis of the cellular responses at 20 days post‐prime (d20), and 7‐ or 21 days post‐boost (d21 + 7 and d21 + 21, respectively). (B) Antigen‐specific CD8^+^ T cells were stained with a K^b^/S_539–546_ tetramer, and the frequencies of K^b^/S_539–546_^+^ CD8^+^ T cells were expressed as a percentage of CD8^+^ T cells. (C) The number of K^b^/S_539–546_^+^ CD8^+^ T cells in the spleen. (D–F) Expression of KLRG1^+^CD127^−^ effector phenotype (D), CD62L^−^CD127^+^ effector memory phenotype (E), and CD62L^+^CD127^+^ central memory phenotype (F) were quantified as a percentage of K^b^/S_539–546_^+^ CD8^+^ T cells (Tet^+^ CD8^+^ T cells). All bar graphs show the mean ± SEM. For all bar plots, each dot represents an animal, and data are from the number of mice as specified in (A; *n *= 9–10). Data are pooled from two independent experiments. Experimental groups were compared to each other by Student's *t*‐test; *p < 0.05.

We initially assessed the requirement for CD4^+^ T cell help for the generation of primary CD8^+^ T cell responses to Moderna's mRNA‐1273 vaccine. Anti‐CD4 monoclonal antibody was administered a day prior to immunization to deplete CD4^+^ T cells. C57BL/6J mice were then immunized intramuscularly in the right hind leg with 1 µg of mRNA‐1273, and CD8^+^ T cell responses were assessed at 20 days post‐prime (d20) and 7‐ or 21‐days post‐boost (d21 + 7 and d21 + 21, respectively; Figure 1A). The cellular responses were quantified by staining for antigen‐specific CD8^+^ T cells with the immunodominant K^b^‐restricted S_539–546_ peptide tetramer (K^b^/S_539–546_; Figure S1). Antigen‐specific CD8^+^ T cells were classified as effector (KLRG1^+^CD127^−^), effector memory (CD62L^−^CD127^+^), or central memory (CD62L^+^CD127^+^).

Given that studies have found a model‐dependent requirement for CD4^+^ T cell help for primary CD8^+^ T cell responses but a universal need for the secondary response, antigen‐specific CD8^+^ T cell responses were quantified in the context of the CD8^+^ T cell compartment and compared across the three timepoints (Figure 1B). The frequency of antigen‐specific responses in the blood in the CD4^+^ T cell‐depleted mice at d20 or d21 + 7 was not significantly different in magnitude compared to controls, suggesting a similar degree of priming and boosting (Figure 1B). During the secondary contraction phase (d21 + 21), there was a 58% reduction in the frequency of K^b^/S_539–546_^+^ CD8^+^ T cells in the blood of CD4^+^ T cell‐depleted mice compared to untreated controls (*p* = 0.013). However, this phenotype was not observed in the spleen as a percentage of CD8^+^ T cells (Figure 1B) or with regards to absolute number of K^b^/S_539–546_^+^ CD8^+^ T cells (Figure 1C).

Upon probing the differentiation states of the K^b^/S_539–546_^+^ CD8^+^ T cells in response to mRNA‐1273 vaccination, no significant differences were recorded in the effector (Figure 1D), effector memory (Figure 1E), or central memory (Figure 1F) phenotypes across the two groups. As such, despite the slight attenuation of antigen‐specific CD8^+^ T cell responses in the blood 21 days post‐boost in CD4^+^ T cell‐depleted mice, there was no detectable impairment in the potential to differentiate into various effector and memory states. The majority of the CD8^+^ T cell responses post‐prime and boost measured were unimpaired in the absence of CD4^+^ T cells, indicating the redundancy of CD4^+^ T cell help in cellular responses to mRNA‐1273.

### 3.2 Polyfunctionality of CD8^+^ T Cell Responses to mRNA‐1273 Is Also Unimpaired Without CD4^+^ T Cell Help

**FIGURE 2:**
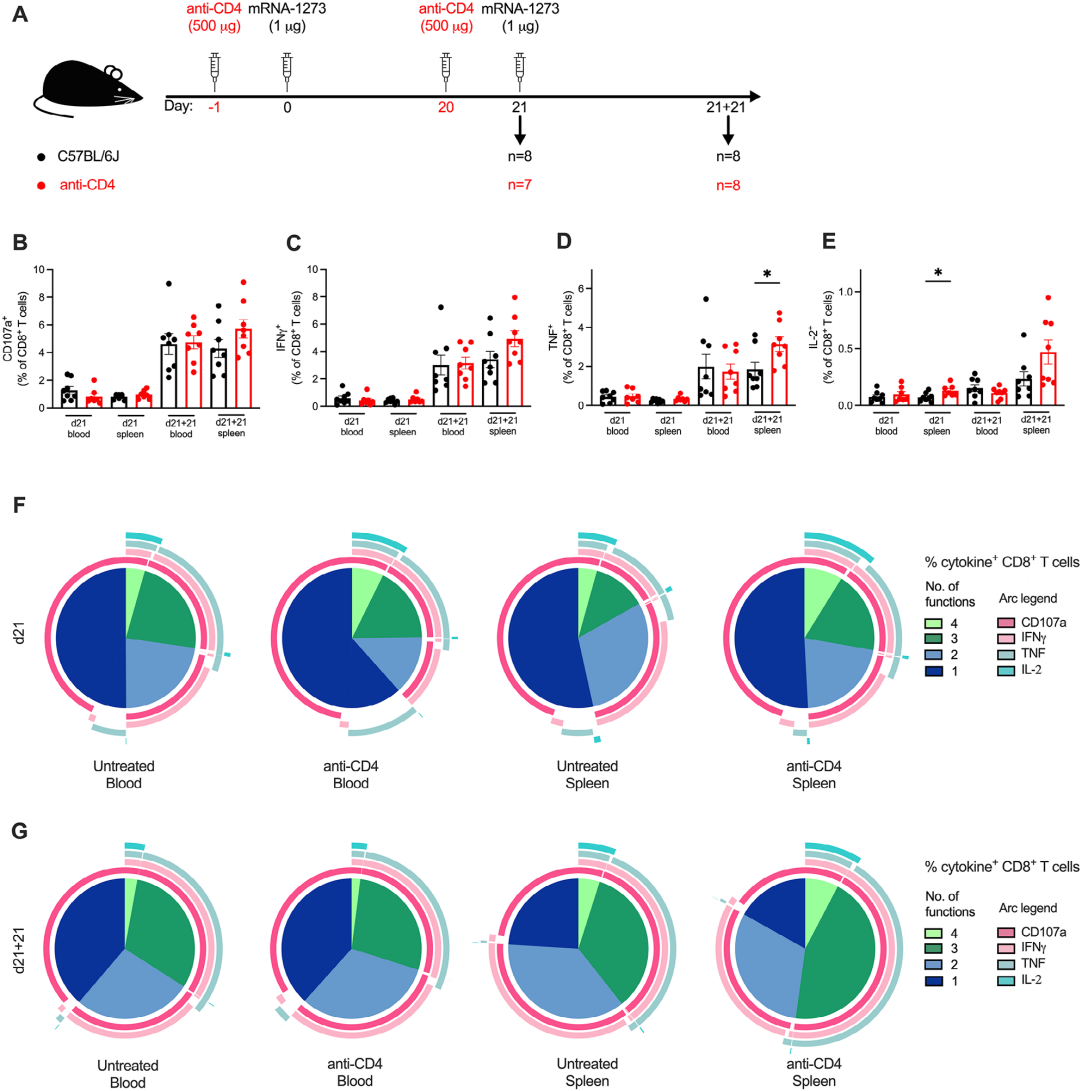
Polyfunctionality of CD8+ T cell responses to mRNA‐1273 is unimpaired without CD4^+^ T cell help. (A) A schematic diagram illustrating the experimental setup: Anti‐CD4 antibody was administered intraperitoneally 1 day prior to each dose of mRNA‐1273, where the boost was given 21 days post‐prime. Cells from blood and spleen were isolated for analysis of cellular responses at 21 days post‐prime and post‐boost (d21 and d21 + 21, respectively). Intracellular cytokine staining after stimulation with the immunodominant K^b^‐restricted S_539–546_ peptide and flow cytometry were performed. (B–E) The frequency of CD107a^+^ (B), IFNγ^+^ (C), TNF^+^ (D), and IL‐2^+^ (E) CD8^+^ T cells was quantified as a percentage of CD8^+^ T cells. All bar graphs show the mean ± SEM. For all bar plots, each dot represents an animal, and data are from the number of mice as specified in (A; *n *= 7–8). Data are pooled from two independent experiments. Experimental groups were compared to each other by Student's *t*‐test; *p < 0.05. (F–G) Polyfunctional cytokine profile at d21 (F) and d21 + 21 (G) of CD107a, IFNγ, TNF, and IL‐2 expression as a proportion of cytokine^+^ CD8^+^ T cells.

Given the essentially unimpaired number and frequency of antigen‐specific CD8^+^ T cell responses to mRNA‐1273 vaccination, we sought to determine if their functionality as measured by expression of cytokines was impacted by the lack of CD4^+^ T cell help. Spike‐specific CD8^+^ T cells were identified by peptide stimulation with the immunodominant K^b^‐restricted S_539–546_ epitope, and the functionality of the CD8^+^ T cell responses was measured by intracellular cytokine staining (ICS) at 21 days post‐prime and post‐boost (Figure 2A; Figure S2).

We first measured the expression of CD107a, a degranulation marker in the blood and spleens of untreated and CD4^+^ T cell‐depleted mice (Figure 2B). Following the depletion of CD4^+^ T cells, we did not observe any reduction in the frequency of CD107a^+^ CD8^+^ T cells. Evidencing the anamnestic potential of these cells, the expression of CD107a^+^ increased by 3.6‐fold in the blood for untreated mice post‐boost compared to their primary responses and this expansion was 5.7‐fold in the blood for the CD4^+^ T cell‐depleted mice.

The expression of IFNγ (Figure 2C) and TNF (Figure 2D) in blood and spleen was not significantly changed post‐prime by CD4^+^ T cell depletion. However, CD4^+^ T cell‐depleted mice had nearly double the frequency of IL‐2^+^ CD8^+^ T cells in the spleen compared to untreated mice (*p* = 0.02), but this was not significant in the blood at d21 (Figure 2E). Similar to CD107a, a second dose of mRNA‐1273 boosted the frequency of IFNγ and TNF expressing CD8^+^ T cells by 4‐fold, even in the absence of CD4^+^ T cell help. IL‐2 expression also doubled upon boosting. In particular, the TNF^+^ CD8^+^ T cell responses in the spleens of CD4^+^ T cell‐depleted mice were boosted to a greater extent, where the frequency of TNF^+^ CD8^+^ T cells was 71% higher in the of CD4^+^ T cell‐depleted mice compared to the untreated controls (*p* = 0.026).

Polyfunctionality of the responding CD8^+^ T cells post‐prime appeared modestly impacted by the absence of CD4^+^ T cells (Figure 2F). Depletion of CD4^+^ T cells resulted in an increased frequency of CD8^+^ T cells that expressed all four cytokines in the blood and spleen. In contrast, there was a higher proportion of monofunctional CD8^+^ T cells in the blood but not the spleen, which were identified to be TNF^+^ CD8^+^ T cells. Proportions of polyfunctional CD8^+^ T cells post‐boost in CD4^+^ T cell‐depleted mice were similar to that of untreated mice (Figure 2G). Interestingly, post‐boost, the proportion of cytokine^+^ CD8^+^ T cells having three or four functions in the spleen of CD4^+^ T cell‐depleted mice was greater than the untreated controls, consistent with the increase in TNF expression (Figure 2D). Altogether, CD4^+^ T cell depletion had little effect on the functionality of primary and secondary CD8^+^ T cell responses induced by mRNA‐1273, and the robust responses demonstrate cellular responses can be boosted without CD4^+^ T cell help.

### 3.3 Antigen‐Specific CD8^+^ T Cell Responses to the BNT162b2 mRNA Vaccine Do Not Require CD4^+^ T Cell Help

**FIGURE 3:**
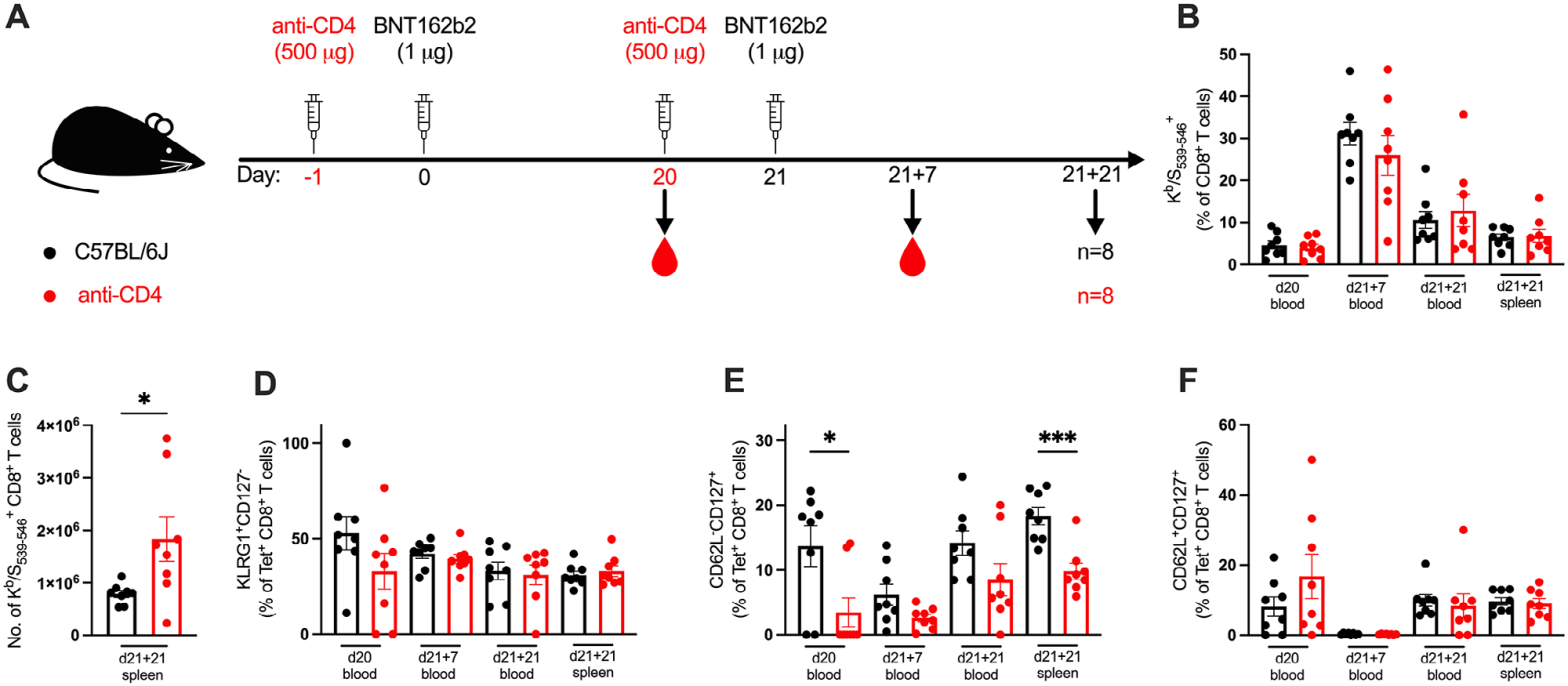
CD4^+^ T cell help is dispensable for generation of antigen‐specific CD8^+^ T cell responses to BNT162b2. (A) A schematic diagram illustrating the experimental setup: Anti‐CD4 antibody was administered intraperitoneally 1 day prior to each dose of BNT162b2. Cells from blood and spleen were isolated for analysis of the cellular responses at 20 days post‐prime (d20), and 7‐ or 21 days post‐boost (d21 + 7 and d21 + 21, respectively). (B) Antigen‐specific CD8^+^ T cells were stained with a K^b^/S_539–546_ tetramer, and the frequencies of K^b^/S_539–546_^+^ CD8^+^ T cells were expressed as a percentage of CD8^+^ T cells. (C) The number of K^b^/S_539–546_^+^ CD8^+^ T cells in the spleen. (D–F) Expression of KLRG1^+^CD127^−^ effector phenotype (D), CD62L^−^CD127^+^ effector memory phenotype (E), and CD62L^+^CD127^+^ central memory phenotype (F) were quantified as a percentage of K^b^/S_539‐546_^+^ CD8^+^ T cells (Tet^+^ CD8^+^ T cells). All bar graphs show the mean ± SEM. For all bar plots, each dot represents an animal, and data are from the number of mice as specified in (A; *n *= 8). Data are pooled from two independent experiments. Experimental groups were compared to each other by Student's *t*‐test; *p < 0.05; ***p < 0.001.

We next sought to determine if the ability to generate CD8^+^ T cell responses independent of CD4^+^ T cell help was consistent across multiple mRNA vaccines. As such, we investigated the CD8^+^ T cell responses to Pfizer‐BioNTech's BNT162b2 vaccine following CD4^+^ T cell depletion (Figure 3A).

CD4^+^ T cell depletion had no impact on the K^b^/S_539–546_^+^ CD8^+^ T cell frequency in the blood after one vaccine dose (d20), at the peak response after two doses (d21 + 7), or at an early memory timepoint (d21 + 21) (Figure 3B). Although no differences in frequency of K^b^/S_539–546_^+^ CD8^+^ T cells were observed in the spleen at d21 + 21, there was a greater absolute number of K^b^/S_539–546_^+^ CD8^+^ T cells in the spleen following CD4^+^ T cell depletion, with 1.83 × 10^6^ cells in the CD4^+^ T cell‐depleted mice and 7.89 × 10^5^ cells in the controls (*p* = 0.029) (Figure 3C).

The proportions of KLRG1^+^CD127^−^K^b^/S_539–546_^+^ CD8^+^ T cells were similar across CD4^+^ T cell‐depleted and control groups (Figure 3D), reflecting CD4^+^ T cell help had little impact on the potential of antigen‐specific CD8^+^ T cells to differentiate into an effector state.

The reliance on CD4^+^ T cell help was more pronounced in the reduced frequency of CD62L^−^CD127^+^ effector memory CD8^+^ T cells (Figure 3E), where there was a 75% reduction in blood at d20 (*p* = 0.019) and a 46% reduction in the spleen at d21 + 21 of CD4^+^ T cell‐depleted mice (*p* = 0.0004). The central memory proportion remained unaffected by the absence of CD4^+^ T cell help across the different timepoints (Figure 3F). Overall, the expansion and differentiation of antigen‐specific CD8^+^ T cells in response to BNT162b2 displayed only minor changes in the absence of CD4^+^ T cell help.

### 3.4 CD8^+^ T Cell Responses to BNT162b2 are Equally Polyfunctional in the Absence of CD4^+^ T Cell Help

**FIGURE 4:**
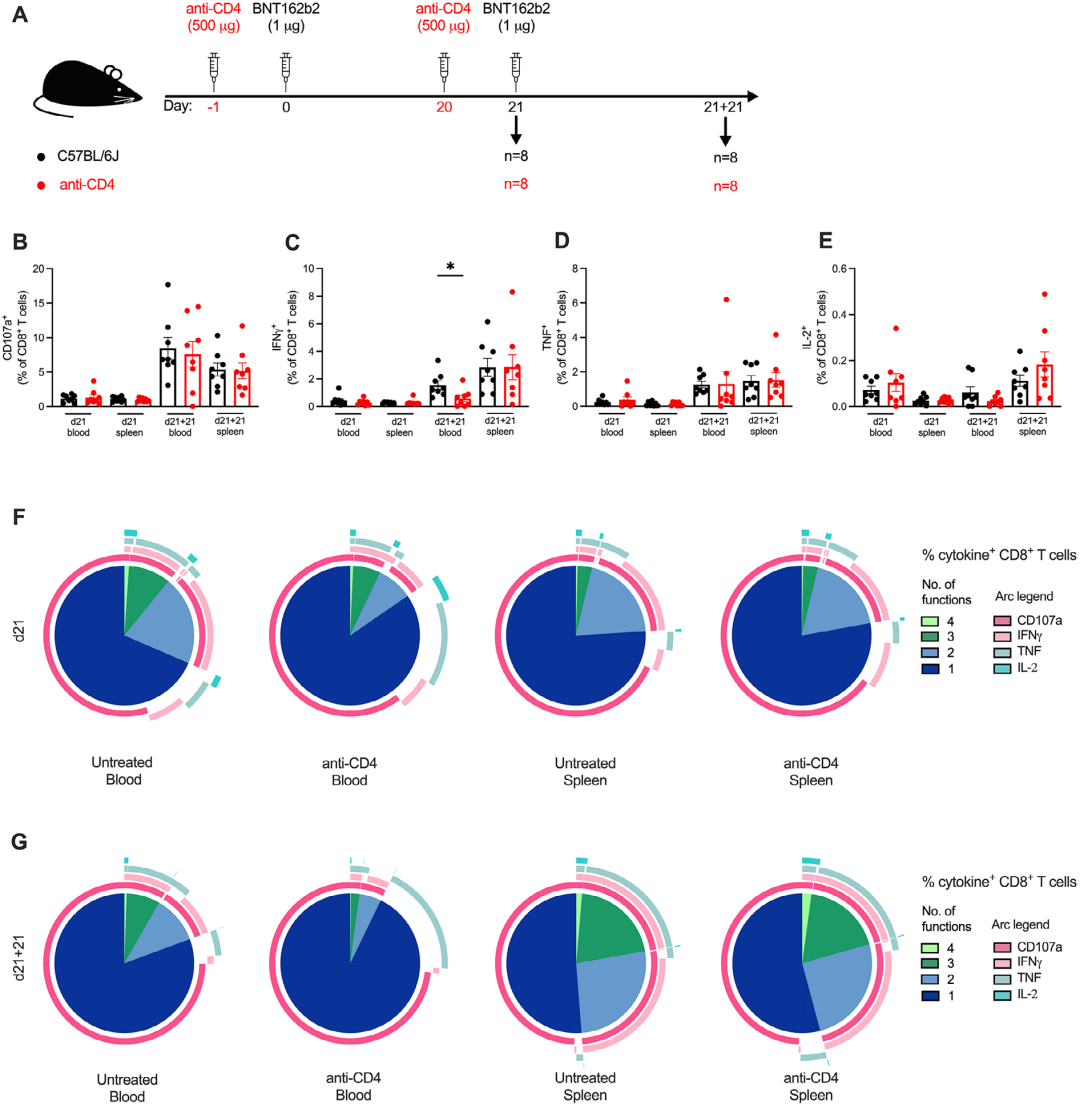
Polyfunctionality of CD8^+^ T cell responses to BNT162b2 is unimpaired without CD4^+^ T cell help. (A) A schematic diagram illustrating the experimental setup: Anti‐CD4 antibody was administered intraperitoneally 1 day prior to each dose of BNT162b2, where the boost was given 21 days post‐prime. Cells from blood and spleen were isolated for analysis of cellular responses at 21 days post‐prime and post‐boost (d21 and d21 + 21, respectively). Intracellular cytokine staining after stimulation with the immunodominant K^b^‐restricted S_539–546_ peptide and flow cytometry were performed. (B–E) The frequency of CD107a^+^ (B), IFNγ^+^ (C), TNF^+^ (D), and IL‐2^+^ (E) CD8^+^ T cells was quantified as a percentage of CD8^+^ T cells. All bar graphs show the mean ± SEM. For all bar plots, each dot represents an animal, and data are from the number of mice as specified in (A; *n *= 8). Data are pooled from two independent experiments. Experimental groups were compared to each other by Student's *t*‐test; *p < 0.05. (F–G) Polyfunctional cytokine profile at d21 (F) and d21 + 21 (G) of CD107a, IFNγ, TNF, and IL‐2 expression as a proportion of cytokine^+^ CD8^+^ T cells.

Similar to mRNA‐1273, the frequency of CD8^+^ T cells expressing each of the cytokines following peptide restimulation of PBMCs or splenocytes from untreated or anti‐CD4 treated mice vaccinated with BNT162b2 was recorded at 21 days post‐prime and post‐boost (Figure 4A).

The expression of CD107a was unaltered in the CD4^+^ T cell‐depleted mice post‐prime or post‐boost (Figure 4B). In fact, the second dose of BNT162b2 led to a >5‐fold increase in blood compared to the first dose, regardless of CD4^+^ T cell depletion. Expression of Th1 cytokines was similarly boosted with the second dose, although to a lesser degree. Minimal differences were observed between the treatment groups in the expression of IFNγ (Figure 4C), TNF (Figure 4D), or IL‐2 (Figure 4E) in primary and secondary CD8^+^ T cell responses. Post‐boost, a reduced expression of IFNγ was observed in the blood of CD4^+^ T cell‐depleted mice at 0.58% compared to 1.56% in untreated mice (*p* = 0.023), although this was not observed in the spleen.

A CD4^+^ T cell‐independent response was reflected in the polyfunctionality, especially in the spleen where there were similar proportions of polyfunctional and monofunctional CD8^+^ T cells (Figure 4F). There was a greater proportion of CD107a^+^ IFNγ^+^ CD8^+^ T cells in the blood of untreated mice than mice depleted of CD4^+^ T cells, although this observation appeared to be heavily influenced by a single mouse with a high frequency of IFNγ^+^ CD8^+^ T cells at d21 + 21 (Figure 4C). This was also observed in the reduced fraction of CD8^+^ T cells with three or four functions in the blood of CD4^+^ T cell‐depleted mice post‐boost (Figure 4G). Nevertheless, the fractions of polyfunctional CD8^+^ T cells in the spleen post‐boost were equivalent in mice depleted of CD4^+^ T cells. Collectively, these data suggest CD4^+^ T cell help is not required for either primary or secondary CD8^+^ T cell responses to nucleoside‐modified mRNA vaccines.

### 3.5 Antigen‐Specific CD8^+^ T Cell Responses to Ad‐Vectored Vaccines Are Dependent on CD4^+^ T Cell Help

**FIGURE 5:**
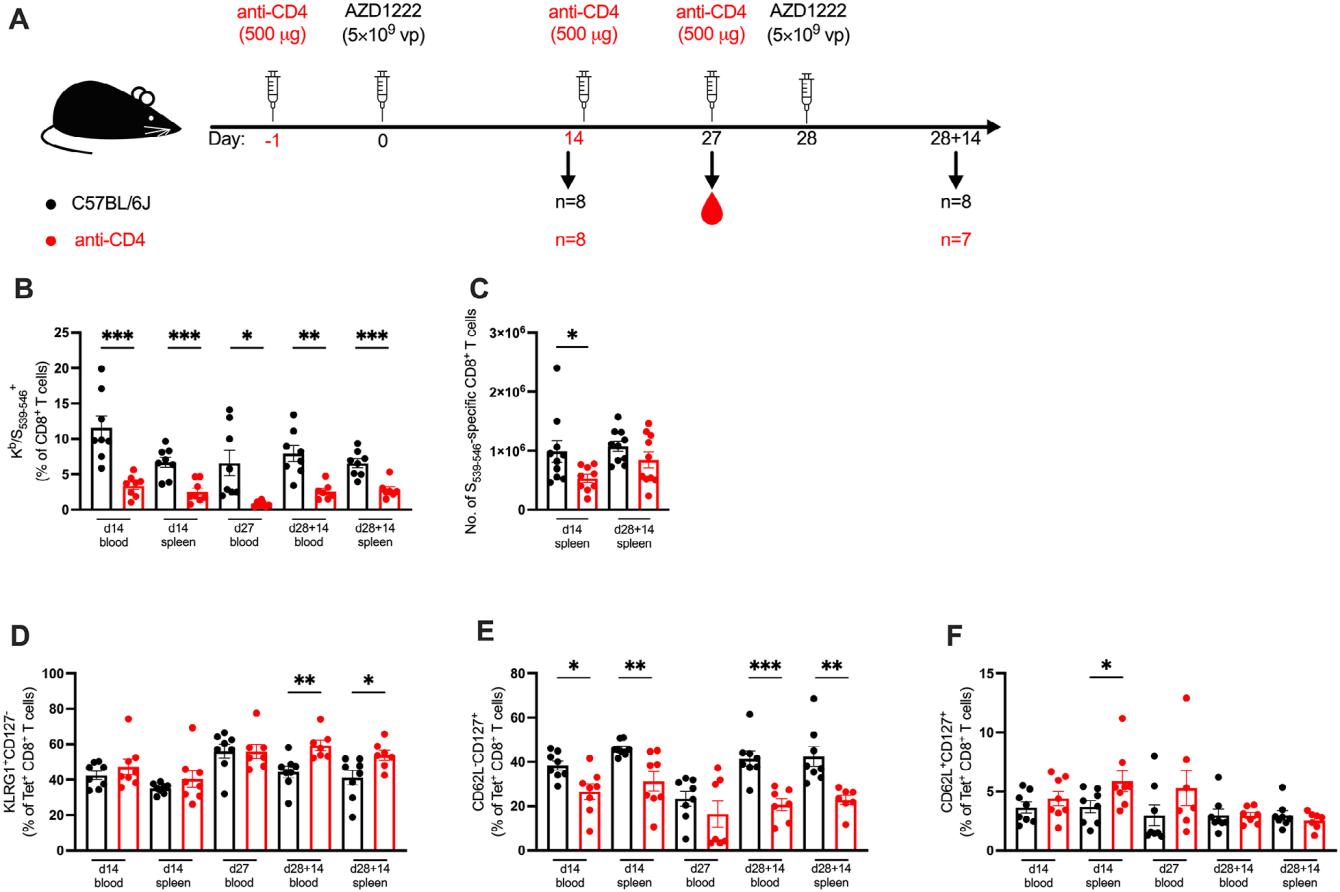
Antigen‐specific CD8^+^ T cell responses to Ad‐vectored vaccines are dependent on CD4^+^ T cell help. (A) A schematic diagram illustrating the experimental setup: Anti‐CD4 antibody was administered intraperitoneally 1 day prior to each dose of AZD1222 and 14 days post‐prime. Animals were then boosted at 28 days post‐prime. Cells from blood and/or spleen were isolated for analysis of the cellular responses at 14‐ or 27 days post‐prime (d14 and d27, respectively), and 14 days post‐boost (d28 + 14). (B) Antigen‐specific CD8^+^ T cells were stained with a K^b^/S_539–546_ tetramer, and the frequencies of K^b^/S_539–546_^+^ CD8^+^ T cells were expressed as a percentage of CD8^+^ T cells. (C) The number of K^b^/S_539–546_^+^ CD8^+^ T cells in the spleen. (D–F) Expression of KLRG1^+^CD127^−^ effector phenotype (D), CD62L^−^CD127^+^ effector memory phenotype (E), and CD62L^+^CD127^+^ central memory phenotype (F) were quantified as a percentage of K^b^/S_539–546_^+^ CD8^+^ T cells (Tet^+^ CD8^+^ T cells). All bar graphs show the mean ± SEM. For all bar plots, each dot represents an animal, and data are from the number of mice as specified in (A; *n *= 7–8). Data are pooled from two independent experiments. Experimental groups were compared to each other by Student's *t*‐test; *p < 0.05; **p < 0.01; ***p < 0.001. Ad, adenovirus.

Given the consistency across the mRNA vaccines, we hypothesized that the redundancy of CD4^+^ T cell help in inducing effector CD8^+^ T cell responses could be an intrinsic trait of the SARS‐CoV‐2 Spike antigen. To assess this, we utilized Oxford‐AstraZeneca's Ad‐vectored vaccine AZD1222 (ChAdOx1 nCoV‐19) to evaluate the cellular responses. Immune responses to AZD1222 were examined at 14‐ and 27 days post‐prime (d14 and d27, respectively), and 14 days post‐boost (d28 + 14) (Figure 5A).

In contrast to the cellular response induced by mRNA immunization, the frequency of K^b^/S_539–546_^+^ CD8^+^ T cells in the CD8^+^ T cell compartment was significantly attenuated across all timepoints in the CD4^+^ T cell‐depleted mice (Figure 5B): 70% in the blood (*p* < 0.001) and 62% in the spleen (*p* < 0.001) at d14; 87% in the blood (*p* = 0.01) at d27; 68% in the blood (*p* = 0.001) and 57% in the spleen (*p* < 0.001) at d28 + 14 in CD4^+^ T cell‐depleted mice. Notably, the K^b^/S_539–546_^+^ CD8^+^ T cell response in the blood of untreated mice was 43% lower at d27 compared to those at d14, suggesting a contraction of cellular responses. Moreover, a second dose of AZD1222 did not significantly boost the frequency of K^b^/S_539–546_^+^ CD8^+^ T cells in the untreated mice. Yet, CD8^+^ T cell response in the blood of CD4^+^ T cell‐depleted mice increased 2.7‐fold from d27 to d28 + 14 when boosted. Given that antigen‐specific CD8^+^ T cells were not completely ablated, this suggests that generation of some effector CD8^+^ T cells in response to AZD1222 could occur without CD4^+^ T cell help. Consistent with this, CD4^+^ T cell depletion significantly reduced the number of primary K^b^/S_539–546_^+^ CD8^+^ T cells at d14, where there was a 46% reduction in the number of antigen‐specific CD8^+^ T cells in the spleen of CD4^+^ T cell mice compared to the untreated controls (*p* = 0.040). This phenotype was reduced, and not statistically significant at d28 + 14 (Figure 5C). Whether this fractional help‐independent response utilizes similar pathways as the CD8^+^ T cell responses to mRNA vaccines or if the reduced antigen‐specific CD8^+^ T cells were sufficient to offer a similar level of protection to those in the untreated animals was not evaluated.

However, the proportion of K^b^/S_539–546_^+^ CD8^+^ T cells expressing KLRG1 in the CD4^+^ T cell‐depleted mice at d14 was similar to that of the untreated group with 47% and 42% KLRG1^+^, respectively, in the blood (*p* = 0.34), and 40.1% and 35.1% KLRG1^+^, respectively (*p* = 0.29), in the spleen (Figure 5D). The expression of KLRG1 in K^b^/S_539–546_^+^ CD8^+^ T cells was greater by 34% (*p* = 0.0046) and 31% (*p* = 0.028) post‐boost in CD4^+^ T cell‐depleted mice blood and spleen, respectively, compared to untreated mice. This suggests that cellular responses without CD4^+^ T cell help did not have a significantly diminished effector phenotype despite the reduced magnitude.

In contrast, there was a more pronounced reduction in the proportion of K^b^/S_539–546_^+^ CD8^+^ T cells expressing a CD62L^−^CD127^+^ effector memory phenotype without CD4^+^ T cell help (Figure 5E). CD4^+^ T cell‐depleted mice had a 31% reduction in CD62L^−^CD127^+^ K^b^/S_539–546_^+^ CD8^+^ T cells in blood (*p* = 0.012) and a 32% reduction in the spleen (*p* = 0.0055) at d14. This was consistent at d28 + 14 with a 49% reduction (*p* = 0.0003) in the blood and a 45% reduction in the spleen (*p* = 0.0016) of CD62L^−^CD127^+^ cells in CD4^+^ T cell‐depleted mice. This reduction was not observed at d27 but could possibly be attributed to a contraction of the effector memory phenotype in the untreated mice at 4 weeks post‐immunization.

The central memory fraction remained largely unperturbed in the absence of CD4^+^ T cell help (Figure 5F) and was significantly greater in the spleen at d14 with 5.88% CD62L^+^CD127^+^ in CD4^+^ T cell‐depleted mice and 3.71% in untreated mice (*p* = 0.049). This was largely driven by one data point that also corresponded to the animal with the lowest frequency of K^b^/S_539–546_^+^ CD8^+^ T cells in the spleen. Furthermore, the overwhelming reduction in frequency of K^b^/S_539–546_^+^ CD8^+^ T cells at boost and effector memory phenotype across the timepoints highlights a requirement for CD4^+^ T cell help in the secondary responses to AZD1222. Overall, these data demonstrate that the generation of antigen‐specific CD8^+^ T cell responses is impaired but not completely ablated in the absence of CD4^+^ T cell help, and CD4^+^ T cell help may be necessary for differentiation into cells with optimal effector memory functions.

### 3.6 Polyfunctionality of CD8^+^ T Cell Responses to AZD1222 Is Perturbed When CD4^+^ T Cell Help Is Lacking

**FIGURE 6:**
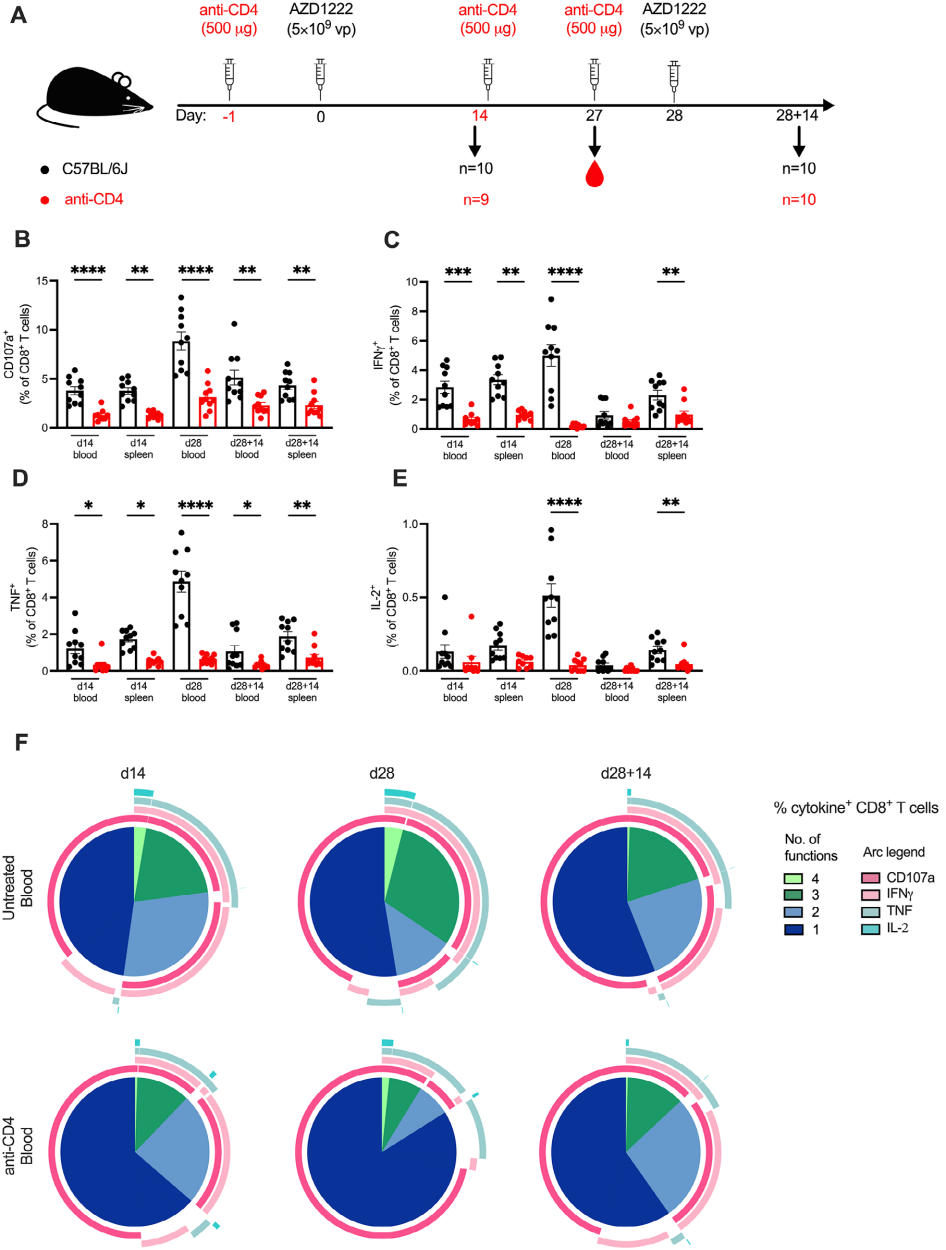
Polyfunctionality of CD8^+^ T cell responses to AZD1222 is perturbed without CD4^+^ T cell help. (A) A schematic diagram illustrating the experimental setup: Anti‐CD4 antibody was administered intraperitoneally 1 day prior to each dose of AZD1222 and 14 days post‐prime. Animals were then boosted at 28 days post‐prime. Cells from blood and/or spleen were isolated for analysis of cellular responses at 14‐ and 27‐days post‐prime, and 14 days post‐boost (d14, d27, and d28 + 14, respectively). Intracellular cytokine staining after stimulation with the immunodominant K^b^‐restricted S_539–546_ peptide and flow cytometry were performed. (B–E) The frequency of CD107a^+^ (B), IFNγ^+^ (C), TNF^+^ (D), and IL‐2^+^ (E) CD8^+^ T cells was quantified as a percentage of CD8^+^ T cells. All bar graphs show the mean ± SEM. For all bar plots, each dot represents an animal, and data are from the number of mice as specified in (A; *n *= 9–10). Data are pooled from two independent experiments. Experimental groups were compared to each other by Student's *t*‐test; *p < 0.05; **p < 0.01; ***p < 0.001; ****p < 0.0001. (F) Polyfunctional cytokine profile of the blood at the different timepoints for CD107a, IFNγ, TNF, and IL‐2 expression as a proportion of cytokine^+^ CD8^+^ T cells.

With the impaired expansion of K^b^/S_539–546_^+^ CD8^+^ T cell responses to AZD1222 vaccination, we sought to elucidate if antigen‐specific expression of cytokines and polyfunctionality were equally impaired in the absence of CD4^+^ T cell help. As such, we performed ICS at 14‐ and 27‐days post‐prime (d14 and d27, respectively) and at 14 days post‐boost (d28 + 14) on mice with continuous CD4^+^ T cell depletion (Figure 6A).

Parallel to the lower frequencies and number of K^b^/S_539–546_^+^ CD8^+^ T cells recorded in CD4^+^ T cell‐depleted mice, these mice had reduced expression of CD107a in the CD8^+^ T cells by >50% at all three timepoints as compared to untreated controls (Figure 6B). Interestingly, there was an increase in CD107a expression at d27 in both untreated and CD4^+^ T cell‐depleted mice compared to d14, but the relatively reduced frequency in CD4^+^ T cell‐depleted mice was maintained (65% reduction; *p* < 0.0001). This opposes the contraction of K^b^/S_539–546_^+^ CD8^+^ T cell frequencies at d27 previously observed (Figure 5B), suggesting that some expression of CD107a can be maintained independently of CD4^+^ T cell help.

The expression of IFNγ was also attenuated in the absence of CD4^+^ T cell help across the timepoints measured, except in the blood at d28 + 14 (Figure 6C). Still, given the 57% reduction in IFNγ^+^ CD8^+^ T cells in the spleens of mice without CD4^+^ T cells compared to untreated mice at this timepoint (*p* = 0.0039), it suggests dependence of IFNγ expression on CD4^+^ T cell help. In contrast to the expression of CD107a at d27, the frequency of IFNγ^+^ in CD4^+^ T cell‐depleted mice was further reduced from 0.96% IFNγ^+^ at d14 to 0.20% IFNγ^+^ at d27, resulting in the most pronounced reduction of IFNγ expression by 96% (*p* < 0.0001) when compared to untreated mice.

Similarly, the expression of TNF was consistently impaired in CD4^+^ T cell‐depleted mice in both blood and spleen at all three timepoints (Figure 6D). This again was most pronounced at d27 where the CD4^+^ T cell‐depleted mice had relatively constant frequencies of TNF^+^ CD8^+^ T cells from d14 to d27, but untreated mice saw a 2.8‐fold increase in frequency of TNF^+^ CD8^+^ T cells in this interval. There was an overall pattern of reduced IL‐2 expression across all timepoints and tissues in CD4^+^ T cell‐depleted mice (Figure 6E), which was statistically significant at d27 (92% reduced; *p* < 0.0001) and at d28 + 14 in the spleen (68% reduced; *p* = 0.0033).

Polyfunctionality of the antigen‐specific CD8^+^ T cells was impaired across the timepoints as evident in the blood (Figure 6F). The fraction of antigen‐specific CD8^+^ T cells expressing 2 or more functions was consistently lower in the CD4^+^ T cell‐depleted mice. Parallel to the individual cytokine expression, this was the most obvious at d27 where the proportion of CD8^+^ T cells expressing 2 or more cytokines in CD4^+^ T cell‐depleted mice was less than half of that in untreated mice. This may indicate an earlier contraction of CD8^+^ T cell functionality when lacking CD4^+^ T cells, especially when compared to an increased polyfunctionality of CD8^+^ T cells that received CD4^+^ T cell help. This, in turn, hints at a role of CD4^+^ T cell help in the maintenance of CD8^+^ T cell function beyond the initiation of CD8^+^ T cell responses to AZD1222.

### 3.7 CD4^+^ T Cell Help Is Essential in the Induction of Antibody Responses Irrespective of Vaccine Platforms

Given that CD4^+^ T cell help was consistently dispensable for the generation of CD8^+^ T cell responses to either mRNA vaccine yet essential for responses to the Ad‐vectored vaccine, we investigated the requirement for CD4^+^ T cell help in the induction of humoral responses.

**FIGURE 7:**
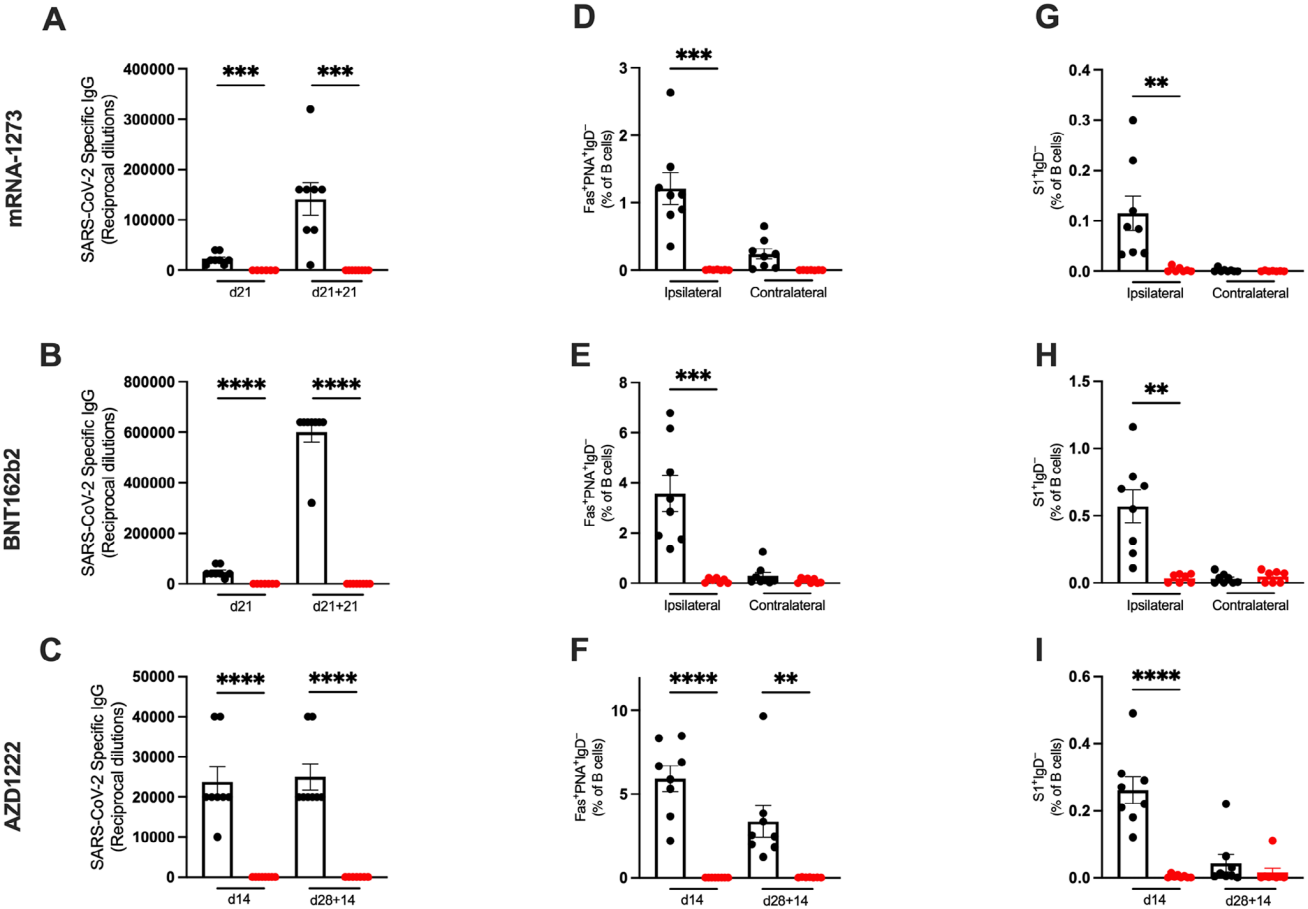
CD4^+^ T cell help is essential in the induction of antibody responses irrespective of vaccine platforms. (A–C) Antibody responses in the sera were quantified as reciprocal dilutions by anti‐spike IgG ELISA post‐prime and post‐boost for each vaccine: d21 and d21 + 21 for mRNA‐1273 (A) and BNT162b2 (B), and d14 and 28 + 14 for AZD1222 (C). (D–I) Cells from inguinal lymph nodes were processed and stained for flow cytometry. The frequencies of GC B cells (Fas^+^ PNA^+^ IgD^−^) following one dose of mRNA‐1273 (D) and BNT162b2 (E) or one and two doses of AZD1222 (F) were quantified as a percentage of B cells. The frequency of spike‐specific (S1^+^) B cells in response to mRNA‐1273 (G), BNT162b2 (H), and AZD1222 (I) was quantified similarly. All bar graphs show the mean ± SEM. For all bar plots, each dot represents an animal, and data are pooled from two independent experiments (*n *= 7–8). Experimental groups were compared to each other by Student's *t*‐test; **p < 0.01; ***p < 0.001; ****p < 0.0001. ELISA, enzyme‐link immunosorbent assay; GC, germinal center.

We first assessed the spike‐specific IgG antibody titers at 21 days post‐prime (d21) and 21 days post‐boost (d21 + 21) with the mRNA vaccines mRNA‐1273 (Figure 7A) and BNT162b2 (Figure 7B); and at 14 days post‐prime (d14) and post‐boost (d28 + 14) with the Ad‐vectored vaccine AZD1222 (Figure 7C). Unlike the cellular responses, the generation of spike‐specific antibodies by all vaccines was fully dependent on CD4^+^ T cell help, and antibodies against spike were completely ablated in the absence of CD4^+^ T cells. This was consistent across both the primary and boosting immunization.

This suggested an impairment in GC formation required for the production of spike‐specific antibodies. To confirm this, we assessed the GC B cell responses (Fas^+^ PNA^+^ IgD^−^ B cells) in the draining inguinal lymph node (Figure S3). As expected, GC B cells were almost absent in the ipsilateral right lymph node of mice without CD4^+^ T cells: 0.007% of B cells in mRNA‐1273 at d21 (Figure 7D), 0.004% in response to BNT162b2 at d21 (Figure 7E), 0.004% to AZD1222 at d14 and 0.002% at d28 + 14 (Figure 7F). In contrast, untreated mice had GC B cell responses to mRNA‐1273 (1.21%), BNT162b2 (3.58%), and to one dose (5.93%) or two doses (3.37%) of AZD1222, mirroring the trends in the anti‐spike IgG titers. GC B cell responses were limited to the ipsilateral side (right lymph node) and not the contralateral lymph node in both the untreated and CD4^+^ T cell‐depleted mice across the mRNA vaccines, coinciding with the side of vaccination (right hind leg). This was the same for Ad‐vectored vaccines (not shown).

Following the three different vaccination regimens, SARS‐CoV‐2 S1‐binding (S1^+^) B cells were also undetectable in the absence of CD4^+^ T cells. Notably, the same dose (1 µg) of mRNA‐1273 induced a 10‐fold lower frequency of S1^+^ B cells at 0.0031% in mRNA‐1273 (Figure 7G) compared to 0.034% in BNT162b2 (Figure 7H) in the CD4^+^ T cell‐depleted mice. Responses to AZD1222 were similar to mRNA‐1273 in the absence of CD4^+^ T cell help at 0.0037% at d14 but appear to be slightly boosted at 0.015% at d28 + 14 (Figure 7I). Nevertheless, the primary S1^+^ B cell responses in CD4^+^ T cell‐depleted mice were orders of magnitude lower compared to untreated mice. Together with the anti‐spike IgG titers and GC B cell frequencies, this demonstrates that while cellular responses to the SARS‐CoV‐2 vaccines have differing requirements for CD4^+^ T cell help depending on the vaccine platform, the role of CD4^+^ T cell help for the generation of humoral responses is universal across the types of vaccine tested.

## 4 Discussion

In this study, we report a discordant role for CD4^+^ T cell help in the induction, functionality, and maintenance of CD8^+^ T cell responses between mRNA and Ad‐vectored vaccines. CD4^+^ T cell help appeared dispensable for the induction of primary and secondary CD8^+^ T cell responses to mRNA vaccines but was required for the CD8^+^ T cell responses to Ad‐vectored vaccines after both vaccinations. In contrast, there was an absolute need for CD4^+^ T cell help for the induction of antibodies by both platforms.

Induction of primary cellular responses that bypass the requirement for CD4^+^ T cell help was shown in response to LCMV and was associated with strong early Type I IFN responses [15]. This was generalized into a coherent model across pathogens and vaccines in a review by Wiesel and Oxenius [35]. A recent study has also demonstrated a critical role for Type I IFN in the induction of a CD8^+^ T cell response to BNT162b2 [36]. This would potentially fit with the above model developed with LCMV. However, serum levels of Type I IFN proteins were low and transient after vaccination with BNT162b2 and mRNA1273 in mice and humans [36–38]. This is specifically due to the vaccine design where nucleoside modification and removal of double‐stranded RNA prevent activation of TLR3 and TLR7, thereby evading the induction of a strong Type I IFN response [16–18]. Thus, our data suggest that the magnitude of Type I IFN response per se may not be the direct factor in bypassing the need for CD4^+^ T cell help, as previously suggested in the model by Wiesel and Oxenius. Instead, the mRNA vaccines in this study might be utilizing a separate pathway to bypass the need for CD4^+^ T cell help. Further work to understand how this corresponds to Type I IFN model is crucial.

The data from Ad‐vectored vaccine‐induced responses further complicates this previous model of CD4^+^ T cell‐independent responses. CD8^+^ T cell responses were significantly reduced following AZD1222 vaccination in the absence of CD4^+^ T cells, consistent with prior data using Ad5‐ or Ad26‐based vaccines [10–12]. However, ChAdOx1, the vector for AZD1222, has been shown to strongly induce Type I IFN [39], as have multiple other Ad vectors [40, 41]. Thus, if Type I IFN is the factor that allows CD4^+^ T cell‐independent primary responses, one would expect the responses to Ad vectors to behave more like those induced by mRNA vaccines. Furthermore, Type I IFN has been shown to inhibit primary CD8^+^ T cell responses to multiple serotypes of Ad vectors [40, 41], although the role of Type I IFN in ChAdOx1‐based vaccine responses has not been explored. Together with the data from the mRNA vaccines, more detailed investigations are required to review the link between strong Type I IFN responses and the requirement of CD4^+^ T cell help in induction of primary CD8^+^ T cell responses.

On the other hand, the ability to induce secondary CD8^+^ T cell responses without CD4^+^ T cell help has not been demonstrated in any models of infection or immunization. This is reflected in our findings of attenuated secondary responses to AZD1222 vaccination, and even in LCMV or *L. monocytogenes* infection, where help was dispensable for primary responses [13, 14, 24]. However, a clinical study found similar levels of Th1‐cytokine^+^ CD8^+^ T cells in patients with idiopathic CD4 lymphopenia compared to healthy controls after two or three doses of the SARS‐CoV‐2 mRNA vaccines [42], mirroring our results with the mouse model. Moreover, CD8^+^ T cell responses to two doses of mRNA vaccines induced in mice lacking CD4^+^ T cell help were shown to be protective against an *Ectromelia* poxvirus challenge in a lethal mousepox model [43]. A recent pre‐print provides a potential model for help‐dependent cellular responses, where the delayed maturation of effector CD8^+^ T cells in the absence of CD4^+^ T cell help in the primary response is due to persistent antigen in a chronic infection [44]. Given the rapid clearance of mRNA at the site of administration or draining lymph nodes [45], this model may explain the help‐independent cellular responses to mRNA vaccines observed in our acute setting.

A separate study described a different model based on the need for CD4^+^ T cell help at prime to generate the secondary cellular responses to immunization [46], where the phenotype of the primary CD8^+^ T cell responses would determine the secondary responses. Interestingly, the same study showed that attenuated CD8^+^ T cell responses at boost could be rescued by the addition of exogenous IL‐2. Unifying these data, subsequent work established that the provision of CD4^+^ T cell helps at priming potentiated autocrine IL‐2 production by CD8^+^ T cells, which allowed for secondary expansion [47]. We note that the frequency of CD8^+^ T cells expressing IL‐2 in the primary and secondary responses to both mRNA vaccines was unimpaired, and consistently marginally higher in the spleen of mice depleted of CD4^+^ T cells. These data possibly suggest a mechanism for how mRNA vaccine‐induced CD8^+^ T cells bypass the need for CD4^+^ T cell‐derived help signals. Nevertheless, the prior model indicated that licensed APCs were needed to induce autocrine IL‐2 signaling via the CD70‐CD27 signaling axis [48, 49]. Further studies are required to elucidate the mechanisms of the crosstalk between APCs and CD8^+^ T cells in the absence of CD4^+^ T cells, and if these APCs are still licensed without CD4^+^ T cells by the mRNA vaccines.

Despite the variable requirement for CD4^+^ T cell help in CD8^+^ T cell responses, our data confirmed the need for CD4^+^ T cell help for antibody responses, as has been widely established in the clinical study mentioned above [42] along with many vaccine and infection models [1]. A previous paper reported T_FH_ cell‐independent antibody responses to the same mRNA vaccines utilized in this study, but complete depletion of CD4^+^ T cells also resulted in loss of antibodies [31], consistent with our findings. In contrast, another study found that the absence of T_FH_ cells using the same mouse model profoundly impaired the humoral response induced by AZD1222 [29]. Thus, even though the need for CD4^+^ T cell help is absolute for the induction of humoral responses by both vaccine platforms, these contrasting data suggest the mechanisms of help might still be different.

CD4^+^ T cells have been established as central orchestrators of cellular and humoral immune responses, yet the requirement for CD4^+^ T cell help in the induction of CD8^+^ T cell responses is variable across vaccines as shown in this study. In the case of mRNA vaccines, we have demonstrated that CD8^+^ T cells can still respond in the absence of CD4^+^ T cells, and robust expansion in secondary immunization is elicited, potentially through autocrine IL‐2 signaling. This is not the case for Ad‐vectored vaccines, which required CD4^+^ T cell help. In contrast, CD4^+^ T cell help is consistently needed by both vaccine platforms for the antibody responses, ultimately highlighting the divergent requirements for CD4^+^ T cell help between platforms. Given both antibody titers and T cell responses are associated with protective COVID‐19 outcomes [50–52], our results highlight the centrality of CD4^+^ T cell in the vaccine‐induced protective immunity against SARS‐CoV‐2.

## Author Contributions

L.Y. performed all experiments, analyzed the data, and prepared the draft manuscript. C.H. assisted with animal work and training. E.B. provided reagents and advice. P.K. and N.M.P. conceived of, secured funding for, and supervised the project. All authors contributed to the editing and revision of the manuscript.

## Conflicts of Interest

The authors note the following conflicts of interest: N.M.P. has received consulting fees from Infinitopes. P.K. has received consulting fees from UCB, Biomunex, AstraZeneca, and Infinitopes. E.B. consults for AstaZeneca, Roche, and Vaccitech and has patents in ChAdOx1 HBV and HCV vaccines.

### Peer review

The peer review history for this article is available at https://publons.com/publon/10.1002/eji.202451142.

## Supporting information



Supporting Information

## Data Availability

The data that support the findings of this study are available from the corresponding author upon reasonable request.
