# Supplementary material for: Distinct Requirements for CD4+ T Cell Help for Immune Responses Induced by mRNA and Adenovirus‐Vector SARS‐CoV‐2 Vaccines
Source: Eur J Immunol. 2024 Nov 27;55(1):e202451142. doi: 10.1002/eji.202451142 (PMC11739681; doi:10.1002/eji.202451142)
Supplement: Supplementary file 1 — Supporting Information [file EJI-55-e202451142-s001.pdf]

Supplementary information

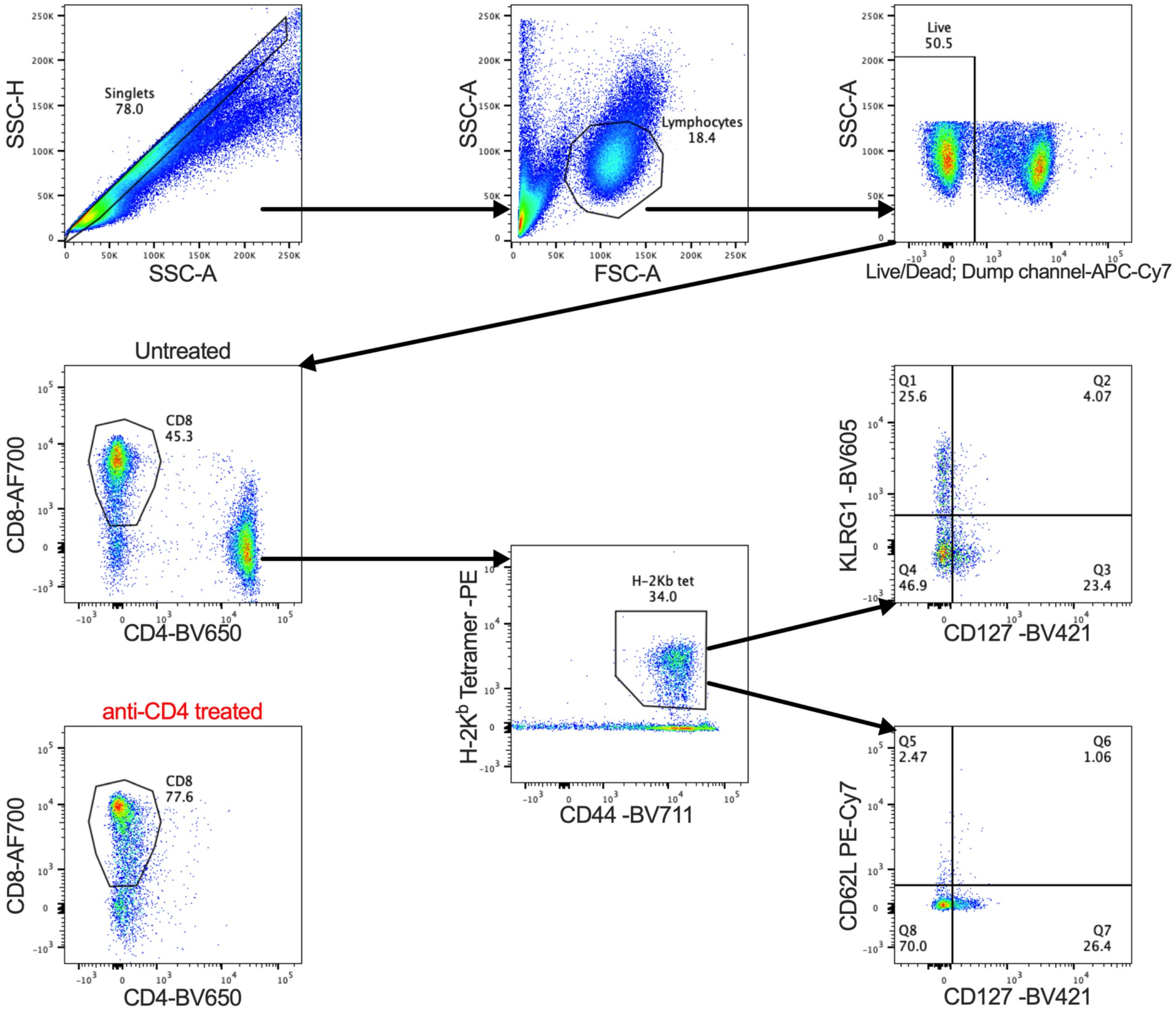

Figure S1. Gating strategy for $K^b/S_{539-546}^+$ $CD8^+$ T cells.

Representative flow plots taken from the spleen 21 days post-boost with mRNA-1273 to show the gating strategy for $Kb/S_{539-546}^+$ $CD8^+$ T cells and KLRG1, CD62L and CD127 expression. Expression of CD4 in the mice administered with anti-CD4 antibody to show the depletion is presented by a single representative flow plot of one mouse sample.

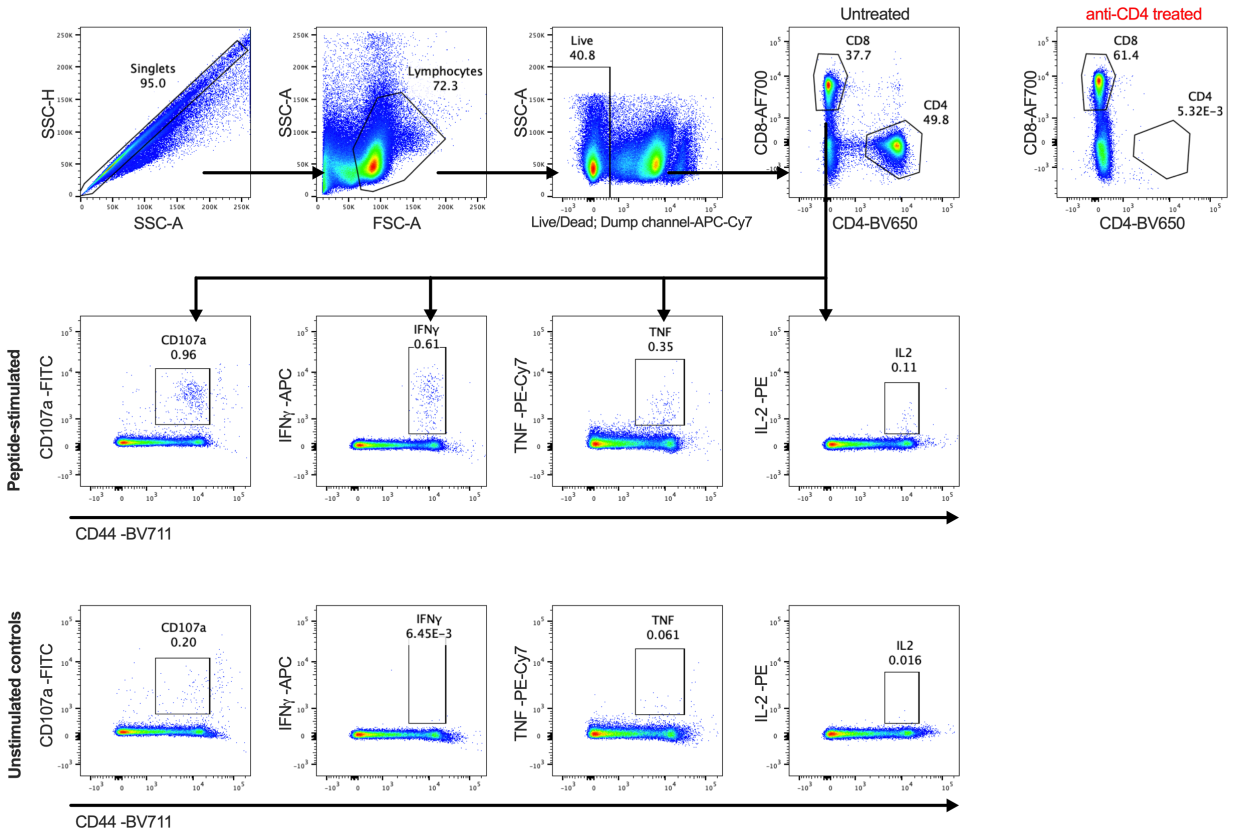

Figure S2. Gating strategy for CD107a and Th1 cytokines on CD8⁺ T cells.

Representative flow plots taken from the spleen 21 days post-prime with mRNA-1273 to show the gating strategy for expression of CD107a, IFNγ, TNF and IL-2 on CD8⁺ T cells after stimulation with the $S_{539-546}$ peptide. Flow plots for those without the peptide stimulation are shown below, as well as a single representative plot of CD4 expression of the CD4⁺ T cell-depleted mice.

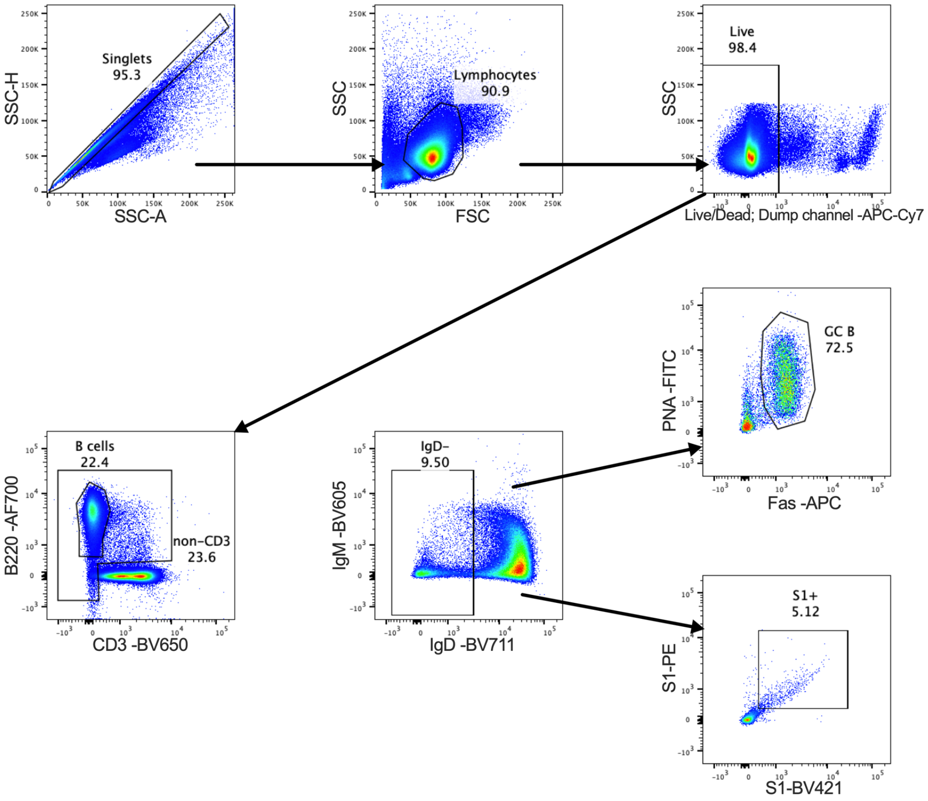

Figure S3. Gating strategy for GC B cells and S1⁺ B cells.

Representative flow plots taken from the ipsilateral (right) inguinal lymph node 14 days post-prime of AZD1222 to show the gating strategy to quantify the frequency of GC B cells and S1⁺ B cells.

Table S1. List of flow cytometry reagents

Primary Reagent	Clone/Catalogue no.	Supplier	Dilution (1 in X)	Conjugate
B220	RA3-6B2	BioLegend	100	AF700, APC-Cy7
Brefeldin A	420601	BioLegend	1000	Purified
CD107a	1D4B	BioLegend	100	FITC
CD127	A7R34	BioLegend	100	BV421
CD138	281-2	BioLegend	100	PE-Cy7
CD3	17A2	BioLegend	50	BV650
CD4	RM4-5	BioLegend	100	BV650
CD44	IM7	BioLegend	100	BV711
CD62L	MEL-14	BioLegend	100	PE-Cy7
CD8a	53-6.7	BioLegend	100	AF700
CD95 (Fas)	15A7	BioLegend	50	APC
F4/80	BM8	BioLegend	100	APC-Cy7
IFN	XMG1.2	BioLegend	100	APC
IgD	11-26c.2a	BioLegend	100	BV711
IgM	RMM-1	BioLegend	100	BV605
IL-2	JES6-5H4	BioLegend	100	PE
KLRG1	2F1	BioLegend	100	BV605
LIVE/DEAD™	L34975	ThermoFisher Scientific	400	Near-IR
NK1.1	PK136	BioLegend	100	APC-Cy7
PNA	FL-1071	Vector Laboratories	50000	FITC
TNF	MP6-XT22	BioLegend	100	PE-Cy7
